# Chemical Compatibility and Electrochemical Performance of Ba_7_Ta_3.7_Mo_1.3_O_20.15_ Electrolytes for Solid Oxide Fuel Cells

**DOI:** 10.3390/ma16113919

**Published:** 2023-05-23

**Authors:** Dong Xu, Xingkai Zhou, Yu Li, Xiaole Yu, Zhexiang Yu, Bochang Shi, Yaowei Mi, Bangze Wu, Lin Ge

**Affiliations:** College of Materials Science and Engineering, Nanjing Tech University, No. 30 South Puzhu Road, Nanjing 211816, China; 202061103074@njtech.edu.cn (D.X.); 202161203206@njtech.edu.cn (X.Z.); 202061103073@njtech.edu.cn (Y.L.); yxl0908@njtech.edu.cn (X.Y.); 202061103052@njtech.edu.cn (Z.Y.); 202161203149@njtech.edu.cn (B.S.); 202161103123@njtech.edu.cn (Y.M.); 202261203315@njtech.edu.cn (B.W.)

**Keywords:** solid oxide fuel cell (SOFC), hexagonal perovskite electrolyte, chemical compatibility, cathode materials

## Abstract

Hexagonal perovskite-related oxides Ba_7_Ta_3.7_Mo_1.3_O_20.15_ (BTM) have recently been reported as promising electrolyte materials for intermediate-temperature solid oxide fuel cells (IT-SOFCs). In this work, sintering properties, thermal expansion coefficient, and chemical stability of BTM were studied. In particular, the chemical compatibilities of (La_0.75_Sr_0.25_)_0.95_MnO_3±δ_ (LSM), La_0.6_Sr_0.4_CoO_3_ (LSC), La_0.6_Sr_0.4_Co_0.2_Fe_0.8_O_3+δ_ (LSCF), PrBaMn_2_O_5+δ_ (PBM), Sr_2_Fe_1.5_Mo_0.5_O_6-δ_ (SFM), BaCo_0.4_Fe_0.4_Zr_0.1_Y_0.1_O_3-δ_ (BCFZY), and NiO electrode materials with the BTM electrolyte were evaluated. The results show that BTM is highly reactive with these electrodes, in particular, BTM tends to react with Ni, Co, Fe, Mn, Pr, Sr, and La elements in the electrodes to form resistive phases, thus deteriorating the electrochemical properties, which has not been reported before.

## 1. Introduction

Solid oxide fuel cells (SOFCs) have attracted much attention as sustainable energy-conversion devices for their high-energy conversion efficiency, fuel flexibility, and environmental friendliness [1]. The electrolyte has been extensively investigated due to its function of connecting the cathode to the anode and conducting ions. Since the operating temperature of Yttria stabilized zirconia (YSZ) is above 700 °C [2], affecting the practical application of the battery, it is necessary to search for an electrolyte that can operate at intermediate temperatures (300–600 °C) with sufficiently high ionic conductivity. Extensive research on crystal structures has led to the discovery of various electrolyte materials with different structures such as the fluorite-type [3,4,5], perovskite-type [6,7,8,9,10], melilite-type [11], and apatite-type [12] structures, that exhibit high ionic conductivity at intermediate temperatures.

Amongst these crystal structures, the perovskite-type structure shows great potential for both oxygen ion conduction and proton conduction due to its flexible framework that can accommodate a variety of cations and its flexible coordination environment that can stabilize oxygen vacancies [13,14,15]. For instance, La_x_Sr_1−x_Ga_1−y_Mg_y_O_3_ (LSGM) exhibits high oxygen ionic conductivity at intermediate temperatures, and doped BaCeO_3_ and BaZrO_3_ showed excellent proton conduction below 600 °C [16,17,18,19].

Recently, the hexagonal perovskite material Ba_7_Nb_4_MoO_20_ was reported to exhibit high ionic conductivity due to its unique disordered structure [20]. Hexagonal perovskite-related structures are composed of hexagonal close-packed AO_3_ (h) layers or sequences of hexagonal and cubic close-packed AO_3_ (c) layers (and anion-deficient AO_3−x_ (c′) where x is the anion vacancy concentration). The disordered stacking of (h) and (c) layers results in the formation of BO_6_ octahedra with face-sharing and corner-sharing [21]. This structure allows the doping of different metal cations to form oxygen vacancies, thus enabling ion transport. Yashima M et al. [22] changed the ratio of cations and thus allowed for the introduction of interstitial oxygen in the (c′) layer, which greatly improved the ionic conductivity of the electrolyte, reaching 5.8 × 10^−4^ S cm^−1^ for Ba_7_Nb_3.9_Mo_1.1_O_20.05_ at 310 °C. Suzuki Y et al. [23] introduced a portion of W^6+^ in the (c′) layer in place of Nb^5+^, which greatly improved the conductivity of Ba_7_Nb_3.85_W_0.15_MoO_20.075_, reaching 2.2 × 10^−2^ S cm^−1^ at 600 °C. Taito Murakami et al. [24] prepared Ba_7_Ta_3.7_Mo_1.3_O_20.15_ (BTM) by replacing Nb^5+^ with Ta^5+^, introduced more interstitial oxygen, with high oxygen ion conduction and only a small amount of electron conductivity, and the conductivity reached 1.08 × 10^−3^ S cm^−1^ at 377 °C.

Therefore, BTM is a promising electrolyte material for SOFC. However, to the best of our knowledge, there is no research available that investigates the chemical compatibility between BTM and electrode materials. During preparation and operation, chemical reactions occur to form new phases due to poor chemical compatibility between materials, which increases the resistance of the cell, hinders the conduction of oxygen ions, and ultimately leads to deterioration of cell performance. Therefore, the selection of electrode materials that match the electrolyte is essential for the performance of cells.

In this work, the thermal expansion coefficient, sintering behavior and electrical conductivity of BTM were characterized. The chemical compatibility of different electrode materials (LSC, LSCF, LSM, BCFZY, PBM, SFM, NiO) [25,26,27,28,29,30] with BTM and the polarization impedance of different materials were investigated by XRD and EIS. In addition, a single cell with Ag|BTM|Ag structure was prepared, and the electrochemical performance of the single cell was tested in the range of 600–800 °C with air as the oxidizer and wet hydrogen (3% H_2_O) as the fuel.

## 2. Experimental

### 2.1. Material Synthesis

The BTM was synthesized using a conventional solid-state reaction method [24]. BaCO_3_, Ta_2_O_5_, and MoO_3_ were weighed with the stoichiometric ratio of Ba_7_Ta_3.7_Mo_1.3_O_20.15_ and ball-milled in ethanol for 24 h. After drying and grinding, the mixture was calcined in air at 1000 °C for 10 h, and the calcined powder was granulated by adding 10% polyvinyl alcohol (PVA). The (La_0.75_Sr_0.25_)_0.95_MnO_3±δ_, La_0.6_Sr_0.4_CoO_3_, and La_0.6_Sr_0.4_Co_0.2_Fe_0.8_O_3+δ_ powders were purchased from Ningbo SOFCMAN Energy Technology Co., Ltd., Ningbo, China. The NiO was purchased from Chengdu Shudu Nanomaterials Technology, Chengdu, China. PrBaMn_2_O_5+δ_ was synthesized by a sol-gel method. Ba(NO_3_)_3_·6H_2_O, Pr(NO_3_)_3_·6H_2_O, Mn(NO_3_)_3_·9H_2_O, and citric acid were dissolved in deionized water, with stirring and heating the solution to obtain the initial powder, then calcined at 1200 °C for 2 h and reduced in wet hydrogen at 800 °C for 2 h to obtain the final powder. The Sr_2_Fe_1.5_Mo_0.5_O_6-δ_ powders were prepared in the same procedure as PBM with Mo_7_(NH_4_)_6_O_24_·4H_2_O, Sr(NO_3_)_2_, and Fe(NO_3_)_3_·9H_2_O as raw materials, and finally calcined at 1100 °C for 5 h. BaCo_0.4_Fe_0.4_Zr_0.1_Y_0.1_O_3-δ_ powders were prepared in the same procedure as BTM, with raw materials BaCO_3_, Co_2_O_3_, Fe_2_O_3_, ZrO_2_, and Y_2_O_3;_ after that, the initial powders were calcined at 1100 °C for 2 h to obtain the final powders.

### 2.2. Preparation of Symmetric Cells and Single Cells

**Figure 1:**
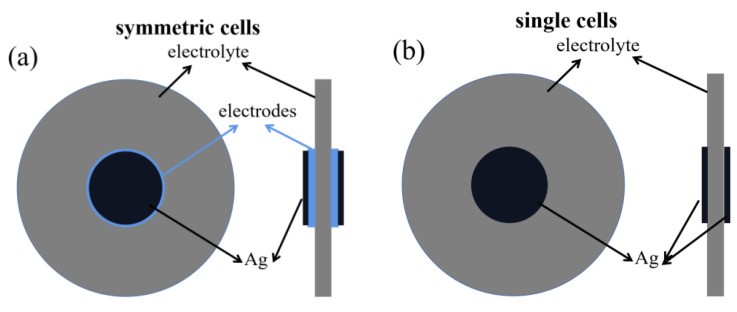
Schematic diagrams of the preparation of (**a**) symmetric cells and (**b**) single cells.

The BTM powder was pressed into pellets under 10 MPa and then sintered in static air at 1300 °C for 4 h. The BTM pellet was used as the electrolyte of the symmetric cell (diameter of 9 mm, thickness of 0.84 mm). A mixture of electrode powder with ethyl cellulose and terpineol in a 1:1.25 mass ratio was used to obtain the electrode ink. The obtained electrode inks were coated on both sides of the BTM electrolyte pellet and then sintered at 1100 °C for 2 h to obtain a symmetric cell. For the preparation of electrolyte-supported single cells, silver paste was coated on both sides of the electrolyte pellet to prepare a single cell with a Ag|BTM|Ag configuration. Figure 1 shows the schematic diagrams of the preparation of symmetric cells and single cells.

### 2.3. Characterization

The phase compositions of the BTM powders were analyzed using X-Ray diffraction (XRD) in the 2θ range of 20–80°. The density of the BTM electrolyte was measured by the Archimedean water displacement method. The surface of electrolyte particles and electrodes are coated with silver paste, which acts as a current collector, and the electrochemical impedance spectra (EIS) of the cells were measured by an electrochemical workstation (CHI 760e, SCHI) at a bias voltage of 5 mV over a temperature range of 450–700 °C and a frequency range of 0.1 Hz to 100 kHz. The effective area of the cell’s cathode was ~0.28 cm^2^.

## 3. Results and Discussion

### 3.1. Characterization of the Ba_7_Ta_3.7_Mo_1.3_O_20.15_ Electrolyte

**Figure 2:**
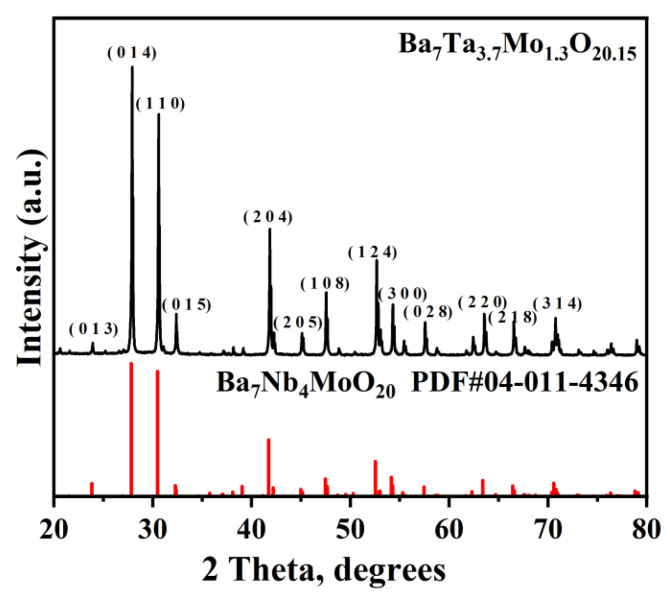
XRD pattern of the BTM powder obtained at 1000 °C for 10 h.

Figure 2 shows the XRD pattern of the BTM powder calcined at 1000 °C for 10 h. The XRD pattern of BTM is similar to Ba_7_Nb_4_MoO_20_, which suggests the successful synthesis of the hexagonal perovskite structure [23,24]. The lattice parameters of BTM are a = b= 5.876 Å and c = 16.589 Å.

**Figure 3:**
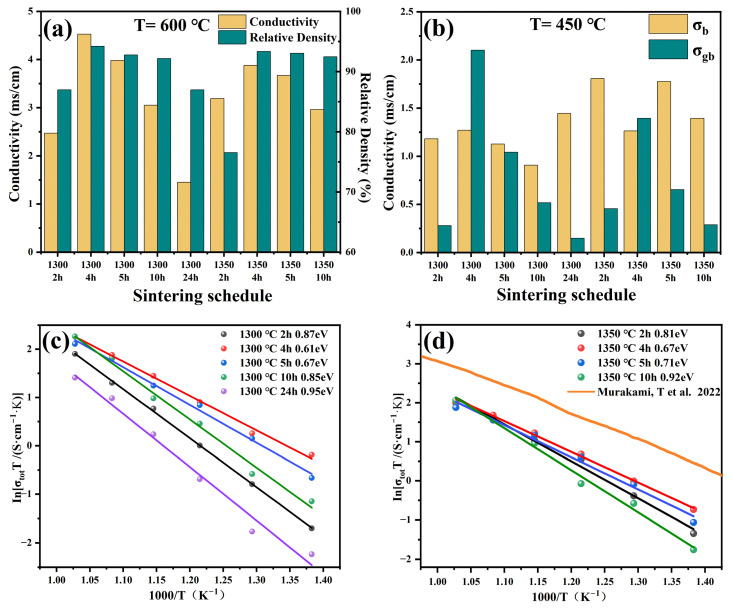
(**a**) Relative densities of electrolytes with different sintering schedules and conductivity measured at 600 °C; (**b**) bulk and grain boundary conductivities of different electrolytes at 450 °C; (**c**) Arrhenius plots of electrolytes sintered at 1300 °C; (**d**) Arrhenius plots of electrolytes sintered at 1350 °C [24].

In order to optimize the sintering process, the physical and electrical properties of the BTM series samples were investigated in relation to the sintering schedule. The BTM powder was pressed into pellets under 10 MPa, sintered at 1300 °C for 2, 4, 5, 10, and 24 h, and at 1350 °C for 2, 4, 5, and 10 h. The relative density and conductivity of the electrolytes with different sintering schedules are given in Figure 3a. When the sintering temperature was 1300 °C, the conductivity and density of BTM increased with the soaking time, and the best performance was achieved when the soaking time was 4 h, and then gradually decreased. When the sintering temperature was 1350 °C, the conductivity and bulk density gradually decreased with the increase in soaking time. The highest density and conductivity of the sample were obtained after sintering at 1300 °C for 4 h. The relative density was 94% and the conductivity measured at 600 °C was 4.5 × 10^−3^ S cm^−1^. Decreased densities at longer soaking times may be attributed to oversintering, which leads to the formation of rapid grain growth and large pores. Furthermore, considering the molybdenum oxide volatilizes at high temperatures, it is reasonable that the conductivity of the BTM electrolyte decreases with increasing temperature and time of sintering. It can be seen from Figure 3b that the density of the electrolyte has a greater effect on the grain boundary conductivity. The grain boundary conductance of dense electrolytes is considerably higher than that of non-dense electrolytes. Figure 3c,d shows the Arrhenius plots of the electrolytes sintered at 1300 °C and 1350 °C, respectively. It is observed that the activation energy decreases with the increase in the electrolyte densities.

**Figure 4:**
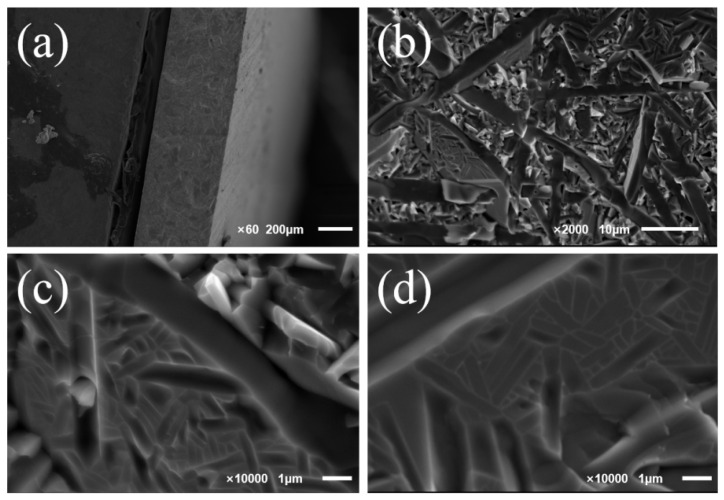
The cross-sectional SEM images of the electrolyte pellets obtained after sintering at 1300 ℃ for 4 h, (**a**) magnification ×60; (**b**) magnification ×2000; (**c**) magnification ×10,000; (**d**) magnification ×10,000.

The SEM images of the cross-section of the electrolyte pellets obtained after sintering at 1300 °C for 4 h are shown in Figure 4. As can be seen in Figure 4a, the electrolytes have a dense microstructure with no obvious pores in the cross-section. It can be noticed from Figure 4c,d that the BTM grains are oblong, which has not been reported before.

**Figure 5:**
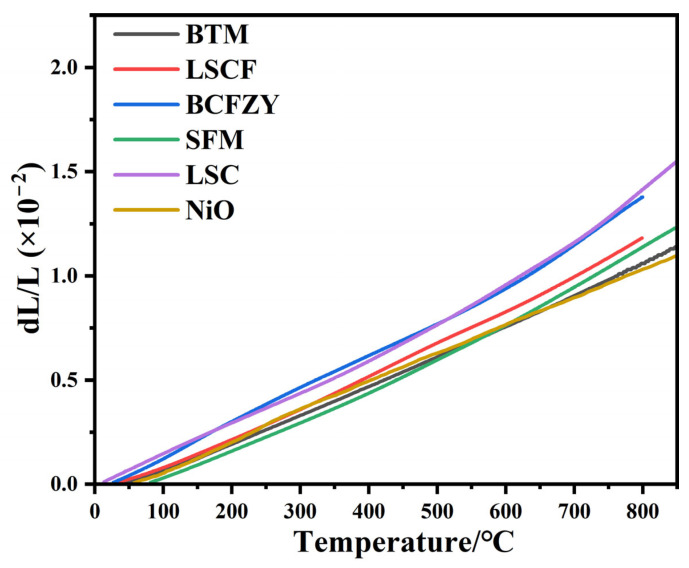
The thermal expansion curves of different materials.

Figure 5 shows the thermal expansion curve (TEC) of BTM, and the TEC (20–800 °C) of BTM is 14.1 × 10^−6^ K^−1^, which is compatible with most electrode materials (15.3 × 10^−6^ K^−1^ for LSCF, 17.2 × 10^−6^ K^−1^ for BCFZY, 14.9 × 10^−6^ K^−1^ for SFM, 20.5 × 10^−6^ K^−1^ for LSC, 14.1 × 10^−6^ K^−1^ for NiO) [31,32,33,34].

### 3.2. Chemical Compatibility of Ba_7_Ta_3.7_Mo_1.3_O_20.15_ with Different Electrodes

The reaction between the electrolyte and the electrode layer is an important issue to be considered when building SOFCs. Considering the variety of electrode materials, several typical electrode materials have been selected to investigate the compatibility with BTM: (a) LSM, which has been studied intensively as the first-generation cathode material, in this work, was examined firstly; (b) Among the developed SOFC electrode materials, Co-based electrode materials have been widely studied for their high mixed conductivity. From the viewpoint of thermal expansion and chemical components, the chemical compatibilities of LSC, LSCF, and BCFZY with BTM were tested; (c) Since the anode is the site of fuel conversion, the compatibility between the anode material and the electrolyte is also essential. The chemical compatibility of BTM with the conventional anode material NiO as well as the perovskite anode materials PBM and SFM were studied.

#### 3.2.1. LSM Electrode

**Figure 6:**
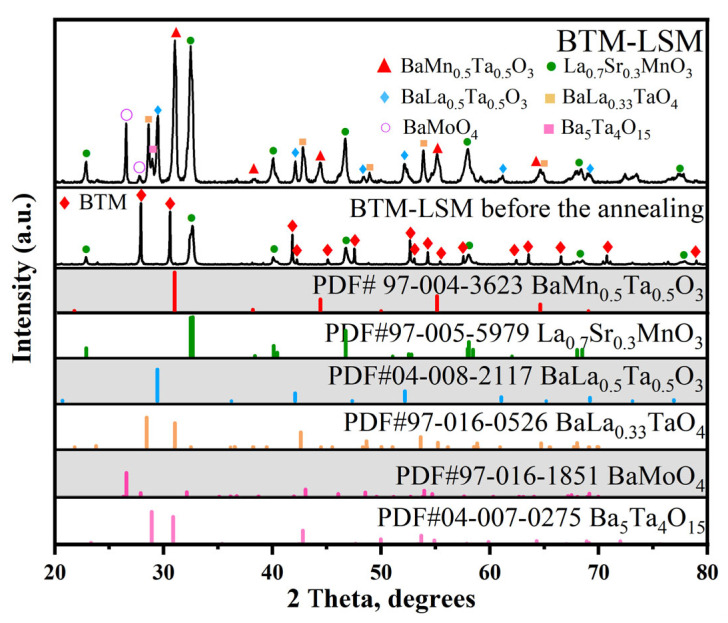
XRD pattern of BTM-LSM mixture (1:1 *w*/*w*) obtained after firing at 1100 °C for 2 h in air.

Figure 6 shows the XRD pattern of the mixture of BTM and LSM after high-temperature heat treatment. It can be seen from the diffraction pattern that the BTM phase has completely disappeared, while the LSM phase still remained. Several other substances were also observed in the diffraction pattern, which were identified as BaLa_0.5_Ta_0.5_O_3_, BaLa_0.33_TaO_4_, BaLa_0.7_Mn_0.3_O_3_, BaMoO_4_, and Ba_5_Ta_4_O_15_. This indicates the high reactivity between BTM and LSM.

#### 3.2.2. LSC, LSCF, BCFZY Electrodes

**Figure 7:**
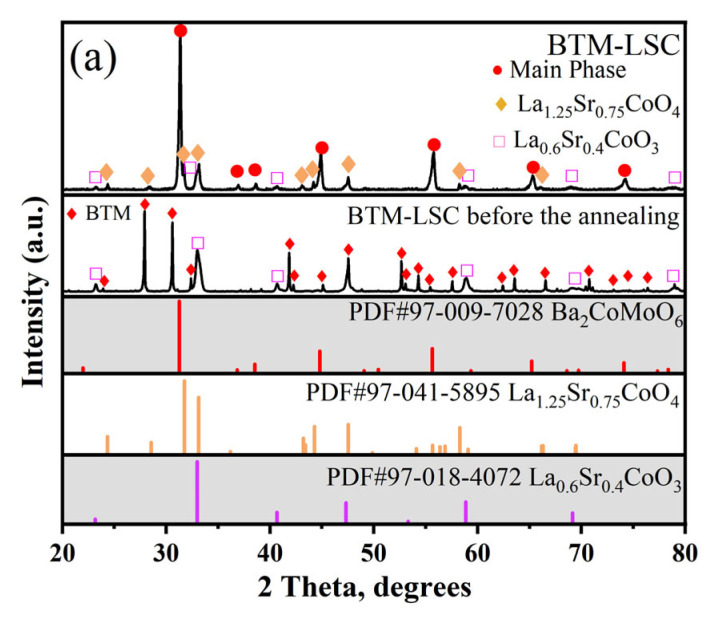

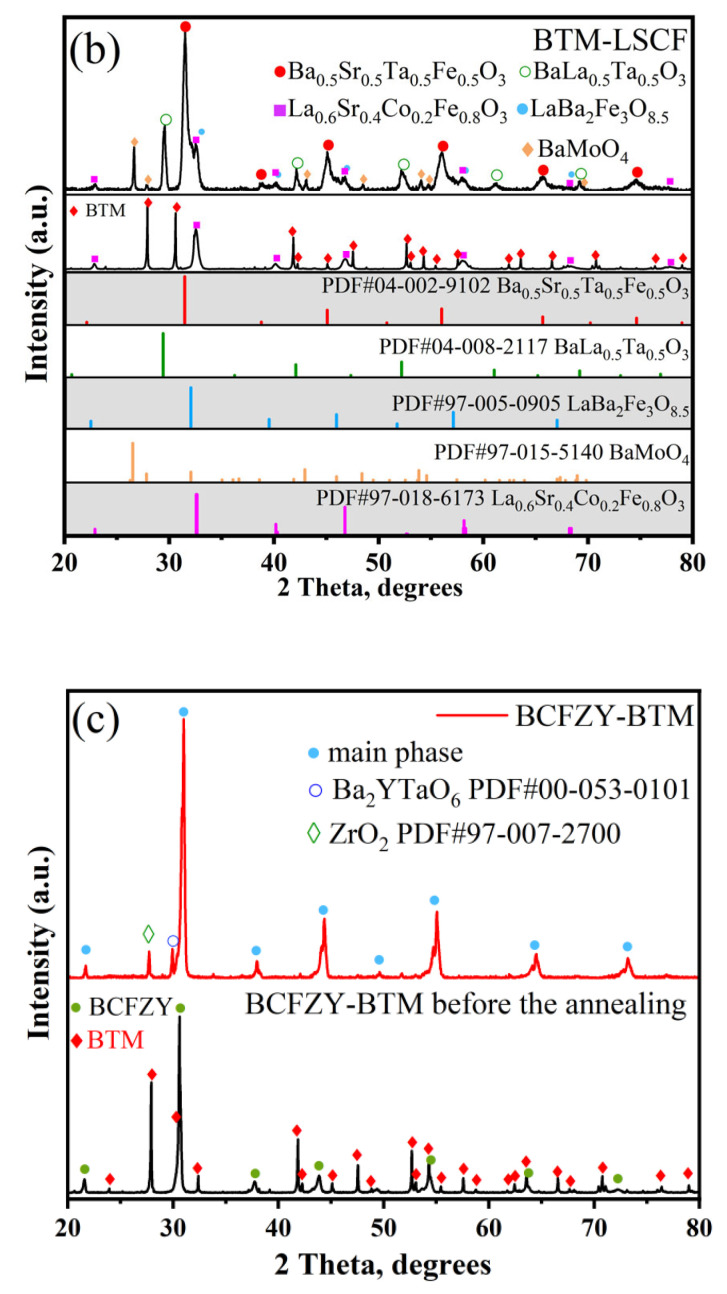
(**a**) XRD pattern of BTM-LSC mixture (1:1 *w*/*w*) obtained after firing at 1100 °C for 2 h in air; (**b**) XRD pattern of BTM-LSCF mixture (1:1 *w*/*w*) obtained after firing at 1100 °C for 2 h in air; (**c**) XRD pattern of BTM-BCFZY mixture (1:1 *w*/*w*) obtained after firing at 1100 °C for 2 h in air.

The XRD patterns of the mixture of BTM and LSC after high-temperature heat treatment are shown in Figure 7a. It can be observed that the main phase shows a perovskite structure and the BTM phase disappears, but the LSC phase still exists, and an impurity phase is indexed to La_1.25_Sr_0.75_CoO_4_ [35]. According to the ICDD database, the main phase peaks can be indexed to Ba_2_MoCoO_6_, and considering that these phases do not contain Ta elements, it is suggested that Ta ions might be doped into the lattice. These results show a high reactivity between BTM and LSC.

The XRD patterns of the mixture of BTM and LSCF after high-temperature heat treatment are shown in Figure 7b. It can be seen from the diffraction pattern that the BTM phase has completely disappeared, while the LSCF phase still exists. Several other phases were also observed in the diffraction pattern, which were identified as the following possible substances: Ba_0.5_Sr_0.5_Ta_0.5_Fe_0.5_O_3_, BaLa_0.5_Ta_0.5_O_3_, LaBa_2_Fe_3_O_8.5_, and BaMoO_4_. This indicates poor compatibility between BTM and LSCF.

The XRD patterns of the mixture of BTM and BCFZY after high-temperature heat treatment are shown in Figure 7c. It is observed that the main diffraction peaks of the calcined mixture are similar to those of BCFZY, and the diffraction peaks of the mixture slightly shift to the high diffraction direction, which might suggest the doping of Ta ions and Mo ions into the lattice. In addition, two small diffraction peaks were observed near 27.7° and 29.9°, which were identified as ZrO_2_ and Ba_3_YFe_2_O_7.5_, respectively.

#### 3.2.3. NiO, PBM, SFM Anodes

**Figure 8:**
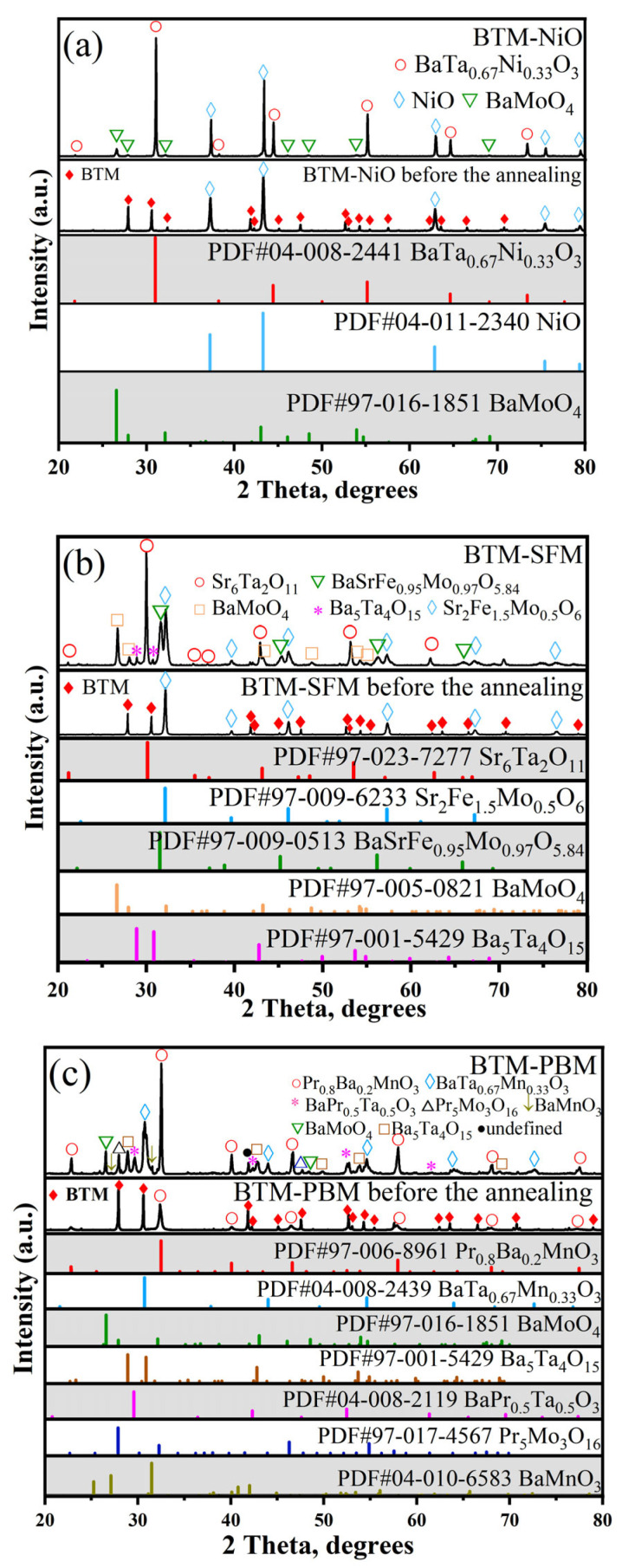
(**a**) XRD pattern of BTM-NiO mixture (1:1 *w*/*w*) obtained after firing at 1100 °C for 2 h in air; (**b**) XRD pattern of BTM-SFM mixture (1:1 *w*/*w*) obtained after firing at 1100 °C for 2 h in air; (**c**) XRD pattern of BTM-PBM mixture (1:1 *w*/*w*) obtained after firing at 1100 °C for 2 h in air.

Figure 8a shows the XRD pattern of the mixture of BTM and NiO after high-temperature heat treatment. It can be observed that the BTM diffraction peaks have disappeared. Three substances are present in the diffraction pattern and are identified as BaTa_0.67_Ni_0.33_O_3_, NiO, and BaMoO_4_. This result indicates the high reactivity between BTM and NiO.

Figure 8b shows the XRD patterns of the mixture of BTM and SFM after high-temperature heat treatment. It is noted that the BTM phase has disappeared, while the SFM phase is still present. Several other substances were also observed in the diffraction pattern, which were identified as Sr_6_Ta_2_O_11_, Ba_5_Ta_4_O_15_, BaSrFe_0.95_Mo_0.97_O_5.84_, and BaMoO_4_. This result implies high reactivity between BTM and SFM. In particular, Mo readily reacts with alkaline earth metal elements, its high reactivity is also observed for other molybdenum-containing materials [27,28].

Figure 8c shows the XRD patterns of BTM and PBM mixture calcined at 1100 °C. It can be seen that the BTM phase has disappeared, while the PBM phase is still present. Several other substances were also detected in the diffraction pattern, which were identified as BaTa_0.67_Mn_0.33_O_3_, Pr_5_Mo_3_O_16_, BaPr_0.5_Ta_0.5_O_3_, Ba_5_Ta_4_O_15_, BaMoO_4_, and BaMnO_3_. This result suggests high reactivity between BTM and PBM. 

In addition, the chemical compatibility of these electrodes with BTM after calcination at 900 °C was investigated, and the results showed that the chemical compatibility of these electrodes with BTM was still poor at relatively low temperatures (Figure S1).

In summary, the XRD analysis results showed low chemical stability between BTM and most electrode materials. Several commonalities in the chemical reactions between BTM and the electrode materials were found:

- After the chemical reaction, the BTM phase usually disappeared completely while the electrode phase remained, more or less;
- BTM may easily react with oxides containing La, Co, Sr, Ni, Mn, Pr, and Fe elements to form new compounds. Unfortunately, most known efficient electrodes, namely mixed ionic-electronic conductor (MIEC), more or less, contain these elements [1,36,37,38,39,40,41,42,43,44,45].

### 3.3. Chemical Stability Analysis

**Figure 9:**
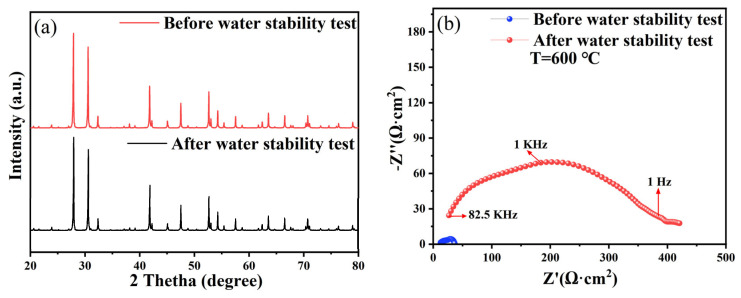
(**a**) XRD patterns of BTM before and after being boiled in water; (**b**) EIS curve of BTM pellets before and after being boiled in water.

The excellent stability of the electrolyte at high-temperatures is important for SOFC, particularly in the presence of water. The excellent stability of BTM in H_2_ has been confirmed [24]. Considering that some Ba-containing electrolytes may precipitate a second phase after soaking in boiling water, the chemical stability of BTM particles in boiling water was tested. The electrolyte pellets were soaked in boiling water at 100 °C for 3 h, and after that, both sides were coated with silver paste for EIS tests and ground into powder for XRD analysis and the results are shown in Figure 9. The XRD pattern shows no significant change in BTM diffraction peaks before and after the water stability test. The EIS spectra showed a significant increase in the ohmic and grain boundary impedance of the electrolyte after the water stability test, which may be due to the generation of a small amount of a second phase on the electrolyte surface. However, the second phase does not appear in the XRD pattern, which may be due to the amount of the second phase being relatively small and exceeding the detection limit of the machine. The pH of the solution increased from 8 to 10 after the water stability test, which proved the poor stability of BTM in high-temperature environments with enriched water.

### 3.4. Electrochemical Impedance Spectroscopy Analysis

**Figure 10:**
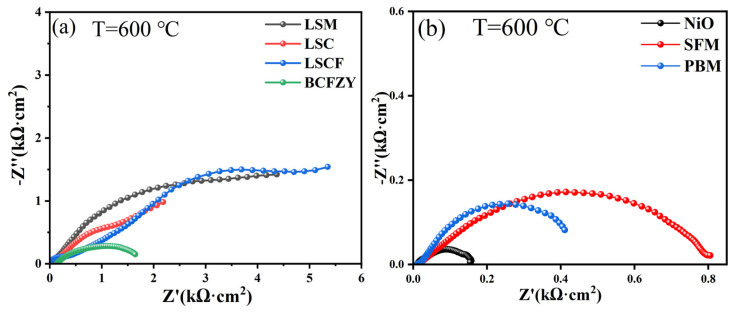
EIS plot of “electrode|BTM|electrode” symmetric cell measured at 600 °C: (**a**) LSM, LSC, LSCF, BCFZY electrodes measured in wet air; (**b**) NiO, SFM, PBM electrodes measured in wet H_2_.

Figure 10a,b shows EIS plots of BTM-based symmetric cells with different electrodes. These electrode materials exhibit particularly poor electrochemical properties. The R_Ω_(R_1_Q_1_)(R_2_Q_2_) equivalent circuit model is used to fit the impedance spectrum, RΩ is the ohmic resistance of the electrolyte and (RQ) corresponds to the different electrode processes. The polarization impedance Rp is the sum of the impedances corresponding to the electrode processes. The polarization impedance values are summarized in Table 1.

As shown in Table 1, the highest polarization impedance of these electrodes is more than 5.31 kΩ·cm^−2^ and the lowest is 0.14 kΩ·cm^−2^. These poor electrochemical performances could be attributed to the aforementioned chemical reactions between BTM and the electrode materials. However, for the electrode of intermediate temperature solid oxide fuel cells, the acceptable ASR values usually do not exceed 1 Ω·cm^−2^. Therefore, the strong chemical reaction between BTM and common MIEC electrodes seriously hinders the application of the BTM electrolyte.

**Figure 11:**
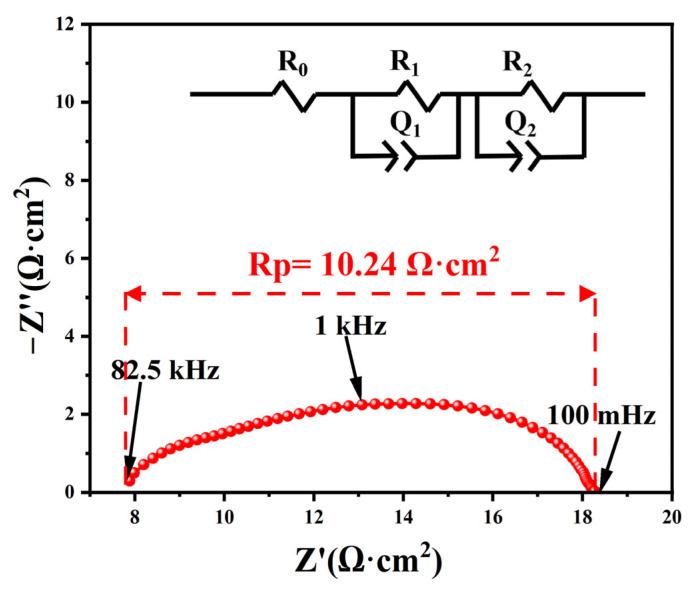
EIS curve of Ag|BTM|Ag symmetric cell.

Figure 11 shows the EIS curve of the Ag|BTM|Ag symmetric cell measured in air at 600 °C. The polarization impedance of silver as an electrode is 10.24 Ω cm^2^. Although silver electrode is still far from offering acceptable performance, it exhibits a relatively low impedance value compared to other electrode materials due to the chemical inertness of silver. In addition, Ag|BTM|Ag single cells were prepared with an open circuit voltage of 1.05 V at 800 °C (Figure S2). This value is close to the theoretical value calculated from the Nernst equation, demonstrating that there is negligible electronic conductivity and few flaws (cracks, pinholes, etc.) in the BTM electrolyte.

## 4. Conclusions

In this paper, the Ba_7_Ta_3.7_Mo_1.3_O_20.15_ electrolyte was prepared by a solid-state reaction. We optimized the sintering regime for BTM, and the conductivity reaches 4.5 × 10^−3^ S cm^−1^ when sintered at 1300 °C for 4 h. The thermal expansion coefficient of BTM was reported for the first time and the average thermal expansion coefficient of BTM is measured as 14.1 × 10^−6^ K^−1^ in the temperature range from 20 to 800 °C. The compatibility of LSM, LSC, LSCF, BCFZY, NiO, SFM, and PBM electrode materials with BTM electrolyte was investigated. Unfortunately, the results of XRD analysis suggested that BTM is very prone to react with electrodes containing La, Co, Sr, Ni, Mn, and Fe elements at high temperatures to form resistive phases and, consequently, result in poor electrochemical performance. This result revealed the high reactivity between BTM and commonly used electrode materials for the first time. Silver, as an electrode, exhibits relatively lower polarization impedance compared to the above materials. Therefore, due to the fact that BTM is not compatible with most known SOFC electrode materials, further research is urgently needed to develop new effective electrodes compatible with BTM or new strategies to alleviate the chemical reactions between BTM and electrodes.

## Figures and Tables

**Table 1:** Polarization impedance values of these electrodes.

Electrode	LSM	LSC	LSCF	BCFZY	NiO	SFM	PBM
Polarization impedance (kΩ·cm^−2^)	>4.26	>2.15	>5.31	~1.49	~0.14	~0.78	~0.4

## Data Availability

All data is available from the corresponding author.

## Supplementary Materials

The following supporting information can be downloaded at: https://www.mdpi.com/article/10.3390/ma16113919/s1. Figure S1: XRD patterns of different materials after firing at 900 ℃ for 2 h in air, (a) BTM-LSM mixture (1:1 *w*/*w*); (b) BTM-LSC mixture (1:1 *w*/*w*); (c) BTM-LSCF mixture (1:1 *w*/*w*); (d) BTM-BCFZY mixture (1:1 *w*/*w*); (e) BTM-NiO mixture (1:1 *w*/*w*); (f) BTM-SFM mixture (1:1 *w*/*w*); (g) BTM-PBM mixture (1:1 *w*/*w*). Figure S2: V-I and P-I curves of Ag|BTM|Ag cell.
