# Supplementary material for: Chemical Compatibility and Electrochemical Performance of Ba7Ta3.7Mo1.3O20.15 Electrolytes for Solid Oxide Fuel Cells
Source: Materials (Basel). 2023 May 23;16(11):3919. doi: 10.3390/ma16113919 (PMC10253820; doi:10.3390/ma16113919)
Supplement: Supplementary file 1 [file materials-16-03919-s001.zip › materials-2372066-SI.pdf]

Chemical Compatibility and Electrochemical Performance of $Ba_7Ta_{3.7}Mo_{1.3}O_{20.15}$ Electrolytes for Solid Oxide Fuel Cells

Dong Xu, Xingkai Zhou, Yu Li, Xiaole Yu, Zhexiang Yu, Bochang Shi, Yaowei Mi, Bangze Wu and Lin Ge *

College of Materials Science and Engineering, Nanjing Tech University, No. 30 South Puzhu Road, Nanjing 211816, China; 202061103074@njtech.edu.cn (D.X.); 202161203206@njtech.edu.cn (X.Z.); 202061103073@njtech.edu.cn (Y.L.); yxl0908@njtech.edu.cn (X.Y.); 202061103052@njtech.edu.cn (Z.Y.); 202161203149@njtech.edu.cn (B.S.); 202161103123@njtech.edu.cn (Y.M.); 202261203315@njtech.edu.cn (B.W.)

* Correspondence: gelin2013@njtech.edu.cn

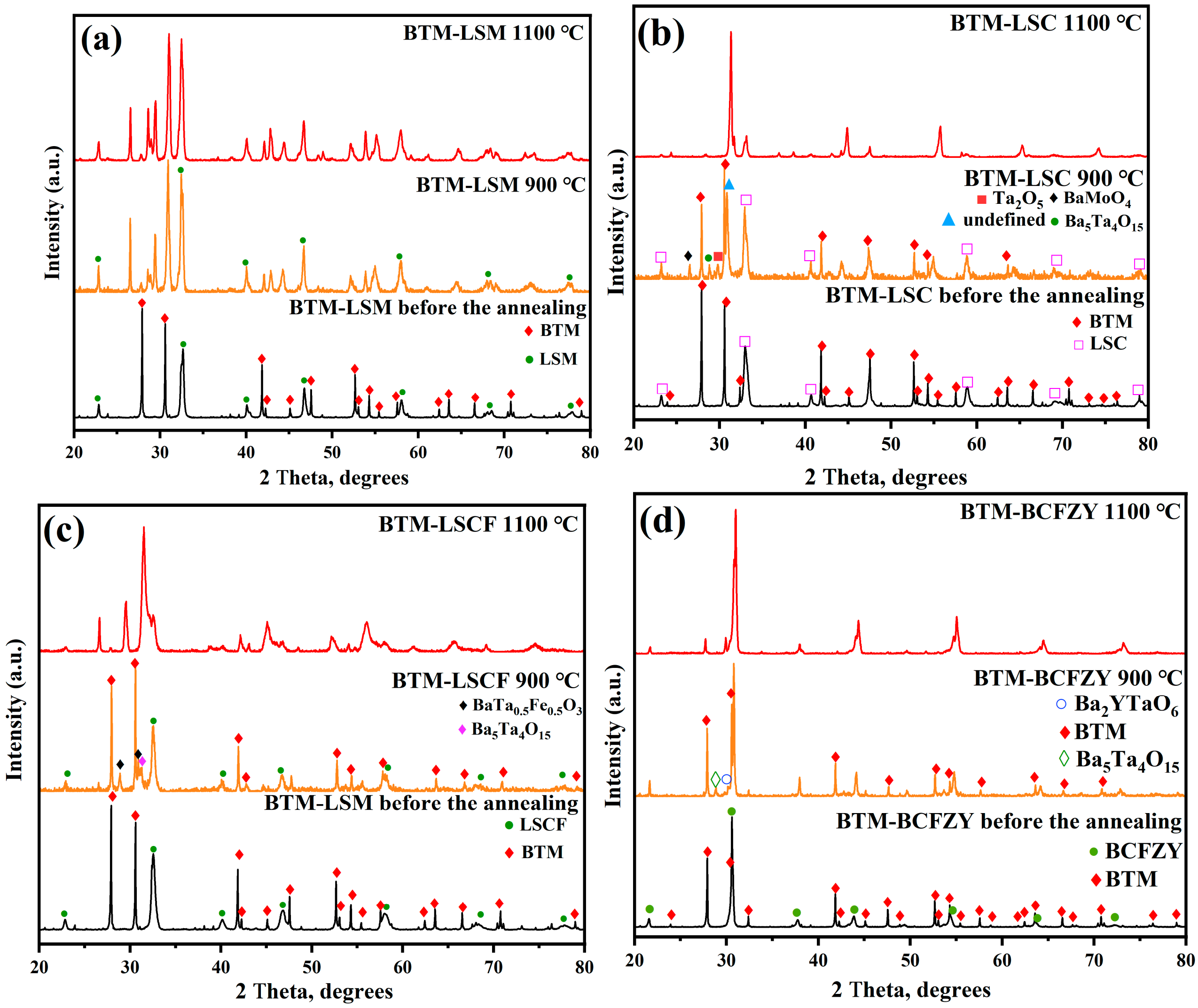

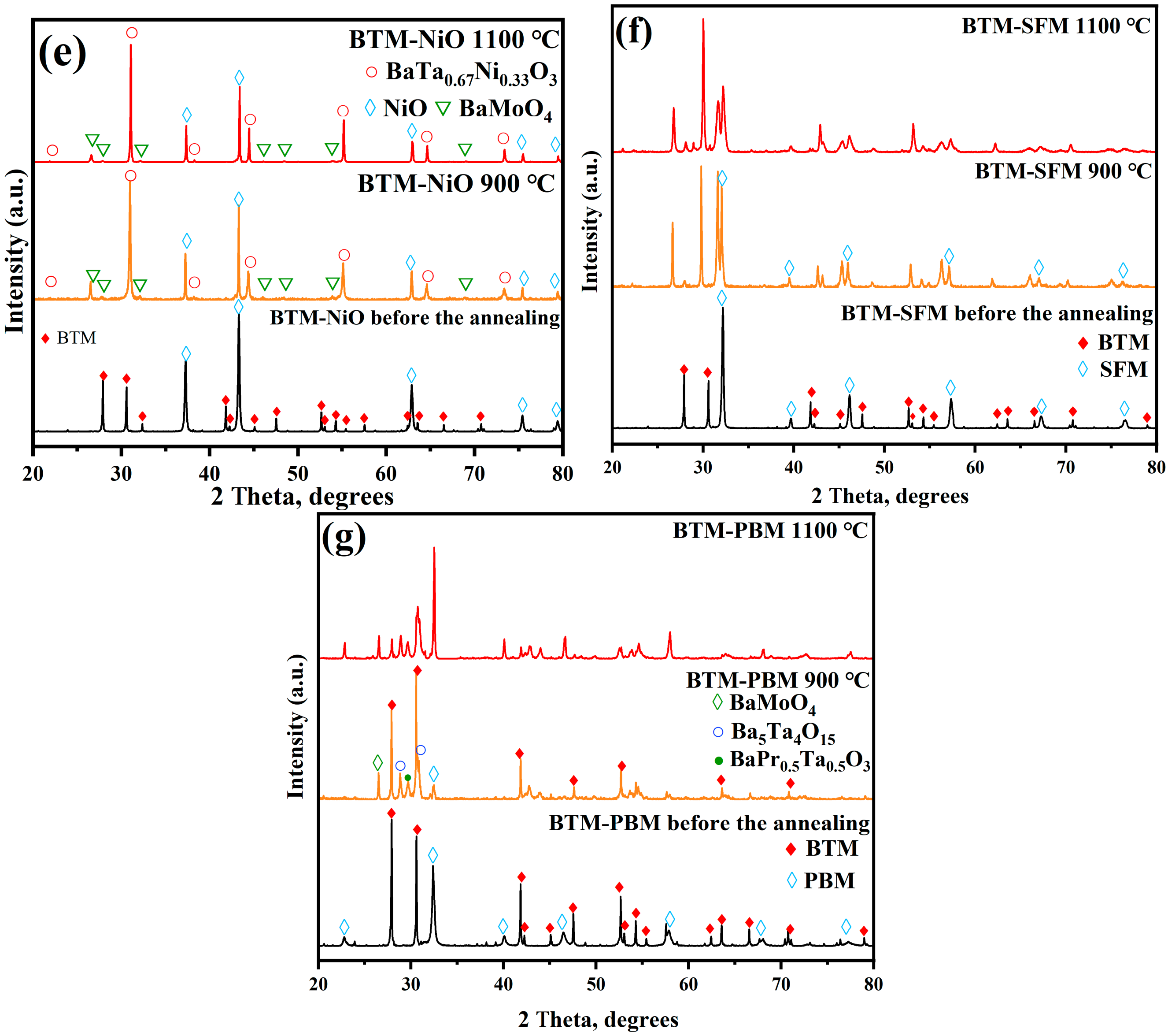

Figure S1. XRD patterns of different materials after firing at 900 ℃ for 2 h in air, (a) BTM-LSM mixture (1:1 w/w); (b) BTM-LSC mixture (1:1 w/w); (c) BTM-LSCF mixture (1:1 w/w); (d) BTM-BCFZY mixture (1:1 w/w); (e) BTM-NiO mixture (1:1 w/w); (f) BTM-SFM mixture (1:1 w/w); (g) BTM-PBM mixture (1:1 w/w).

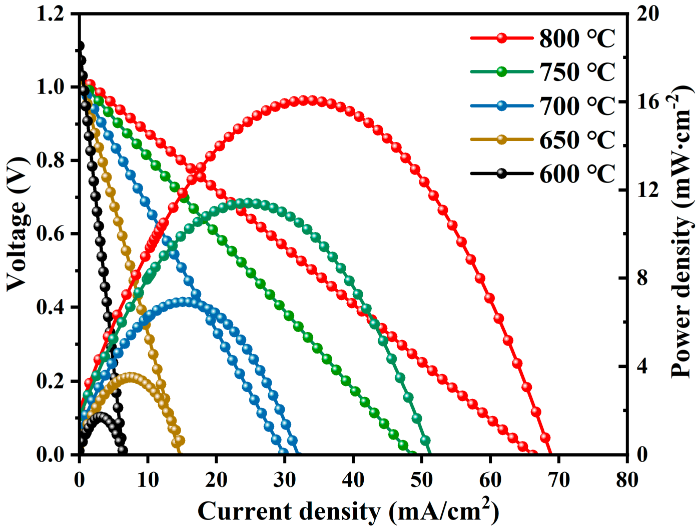

Figure S2. V-I and P-I curves of Ag | BTM | Ag cell.
